# Transfer behavior of odorous pollutants in wastewater sludge system under typical chemical conditioning processes for dewaterability enhancement

**DOI:** 10.1038/s41598-017-03727-4

**Published:** 2017-06-13

**Authors:** Hongyu Gao, Weijun Zhang, Zhenzhen Song, Xiaofang Yang, Lian Yang, Mengdi Cao, Dongsheng Wang, Guiying Liao

**Affiliations:** 1 Faculty of Materials Science and Chemistry, China University of Geosciences, Wuhan, 430074 Hubei China; 2 School of Environmental Studies, China University of Geosciences, Wuhan, 430074 Hubei China; 3 State Key Laboratory of Environmental Aquatic Chemistry, Research Center for co-Environmental Sciences, Chinese Academy of Sciences, Beijing, 100085 China; 4 Technology Center, ZhongYang steel Co., Ltd, Zhongyang, 033400 Shanxi China

## Abstract

Chemical conditioning has been used for enhancing wastewater sludge dewaterability for many years, but the characteristics of odorous pollutants emission in sludge conditioning were still unclear. In this work, the transfer behavior of different odorous pollutants between air, liquid and solid phases under typical chemical conditioning processes for high-pressure dewatering was systematically investigated. The results indicated that that besides hydrogen sulfide (H_2_S) and ammonia (NH_3_), 21 kinds of volatile organic contaminants (VOCs) were identified and quantified by gas chromatography-mass spectrometry (GC-MS), while the concentrations and composition of odorous pollutants varied greatly for different conditioning processes. VOCs were composed by three main constituents including benzenes, halogeno benzene and hydrocarbons. According to mass balance analysis, about 50% of VOCs adsorbed within sludge extracellular polymeric substances (EPS) fraction. Since EPS was damaged and/or flocculation in different chemical conditioning processes, VOCs distributed in solid phase transformed into liquid phase and then released into air. The discrepancies in mass of odorous pollutants before and after chemical conditioning were likely to be related to chemical conversion under acidification, oxidation and precipitation in the presence of ferric ions.

## Introduction

With development of urbanization and growth of population, huge amount of sludge is produced in municipal wastewater treatment. Microorganisms in sludge system are highly active, inorganic salts, organic matters and microorganisms in sewage sludge might break down into a variety of small molecules and volatile gas in the wastewater and sludge treatment process. These gaseous contaminants were easy to release into air environment, thus disturbing healthy and normal lives of residents around wastewater treatment plant (WWTP)^1–3^. Due to differences in each process of wastewater treatment, they differed greatly from types to concentrations of odors^4–6^.

The chemical constituents of odorous contaminants are very complex and primarily consisted of H_2_S, NH_3_ and VOCs. VOCs in WWTP are generally divided into several categories: sulfur compounds, nitrogenous compounds, halogen and its derivatives, hydrocarbons and organic compounds containing oxygen^7, 8^. Hydrogen sulfide and ammonia in odor are not only able to simulate smell, but also seriously corrode equipments in WWTP, consequently shortening the equipment life^9^. In addition, odor has a strong irritant effect to nerve, circulatory, respiratory and endocrine system of bodies^10^. Generally, the concentrations of H_2_S and NH_3_ in air are much higher than that of VOCs, but some of VOCs are believed to be more hazardous than inorganic gaseous pollutants. VOCs would stimulate the body deformities, cancer and genetic mutations, which are the potential and long-term harm to human^11^. It’s well known that odorous pollution from WWTP is one of the seven typical urban pollutions^12^. Odour emissions are considered to be the main cause of disturbance noticed by the citizens living near some facilities. Wang *et al*.^13^ noted that sludge treatment unit is a most important source of odor pollution in whole the WWTP.

It was reported that EPS played an important role in sludge dewatering process^14–16^. In biological wastewater treatment, EPS are produced by the microorganisms in aerobic and anaerobic sludge when organic materials present in wastewater are consumed. EPS are comprised of protein, polysaccharide, humic acid and nucleic acid, in which proteins and polysaccharides are the majority^17^. EPS are considered as the key constituents affecting physicochemical and biological properties, and they are also responsible for sustaining the structural and functional integrity of aggregates. EPS accounts for 60–80% of the mass of waste activated sludge, they play important roles in the removal of pollutants from wastewater, bioflocculation, settling and dewatering of activated sludge^18–20^. According to EPS distribution outside the cells, EPS can be divided into soluble EPS (SEPS) and bound EPS (BEPS)^21–23^. BEPS has rheological double layers including the loosely bound EPS (LB-EPS) and the tightly bound EPS (TB-EPS). LB-EPS plays a decisive role for properties of activated sludge^24^. Organic pollutants in the wastewater were generally removed by EPS sorption through hydrophobic and electrostatic interactions.

High-pressure dewatering processes have been widely used in current China to alleviate the pressure of steadily increasing sludge production. Chemical conditioning is an indispensable step in sludge treatment for dewatering performance enhancement. Organic flocculants were always used in low-pressure dewatering process. Inorganic coagulants were commonly applied as conditioners in high-pressure dewatering process, since their hydrolysis products were to enhance sludge floc strength and reduce sludge compressibility. It was well known that EPS was highly hydrophilic, and traditional chemical flocculants were ineffective to remove the bound water trapped in sludge. Therefore, advanced treatment processes were developed to destroy sludge EPS fractions and convert the bound water within sludge flocs into free water^25, 26^.

Unlike organic flocculants, addition of inorganic conditioners might greatly affect sludge properties and solution chemistry conditions, subsequently resulting in emissions of odorous pollutants. Additionally, hydrophobic organic substances such as VOCs absorbed in sludge was very likely to release with EPS degradation under advanced treatment processes. However, few of studies have examined the transfer behavior of different odorous pollutants under chemical conditioning processes. In this work, the emissions characteristics and mechanisms of odorous contaminants under typical sludge conditioning processes were investigated in details. Especially, various VOCs were identified and quantified based on GC-MS. Finally, the changes in physicochemical properties of sludge system were analyzed to understand their relationships with odorous pollutants.

## Results and Discussion

### Effects of chemical conditioning with different methods on the characteristics of sludge

#### Effects of different chemical conditioning processes on sludge dewatering performance

**Figure 1:**
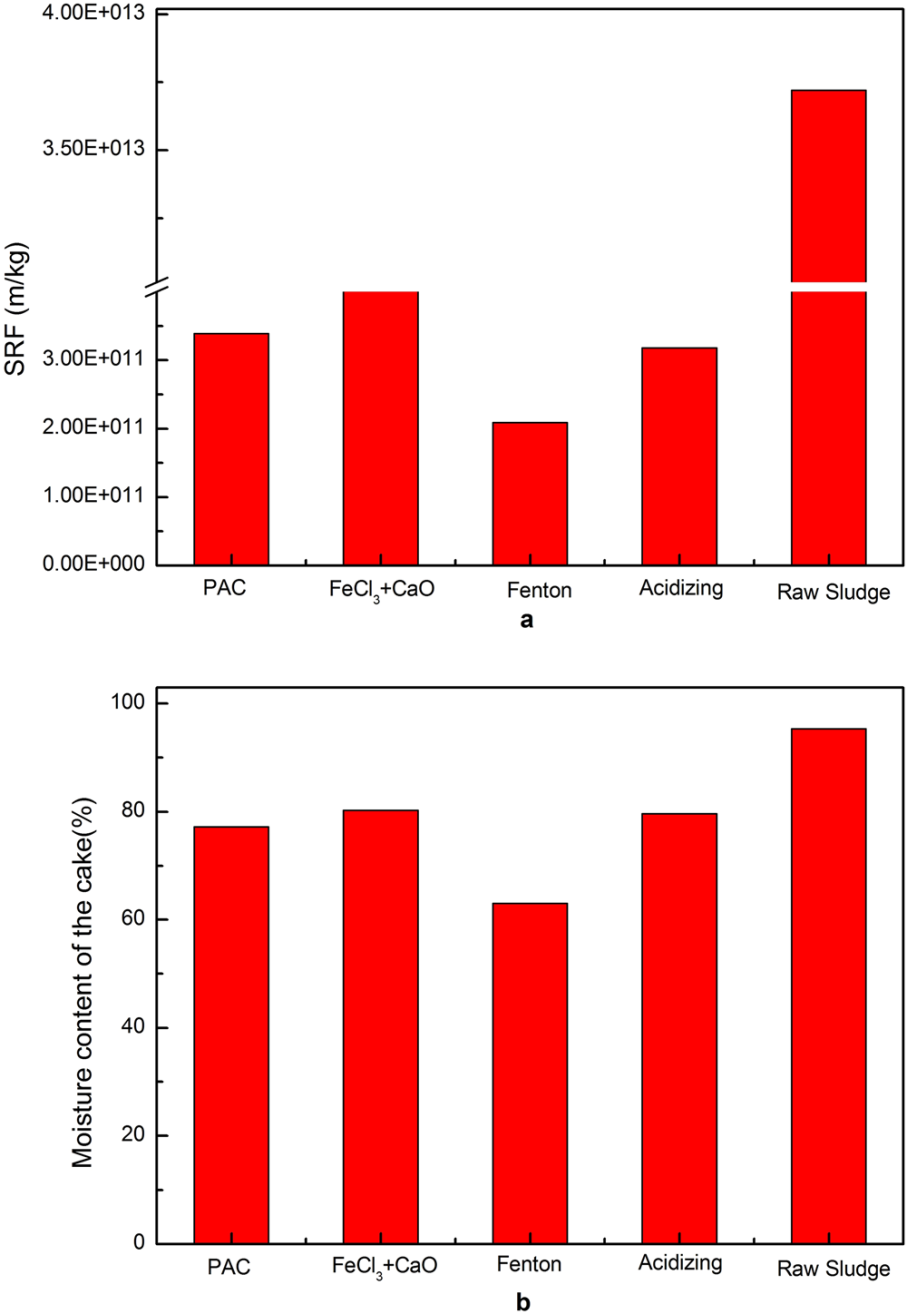
Effect of four inorganic coagulants on SRF (**a**) and moisture content of the cake (**b**).

According to the value of specific resistance to filtration (SRF), sludge can be classified into the sludge with bad dewaterability (>E + 12 – E + 13 m · kg^−1^), medium dewaterability ((5–9) E + 11 m · kg^−1^) and good dewaterability (<4 E + 11 m · kg^−1^)^27^. As described in Fig. 1(a), SRF of raw sludge was 37.21 E + 12 m/kg. Fenton treatment performed better in sludge dewatering improvement than other methods. As can be seen from Fig. 1(b), the moisture content of sludge cake (MC) with typical chemical reagents was as follows: FeCl_3_ + CaO > acidification > PAC > Fenton treatment. It was reported that addition of inorganic coagulants could eliminate the negative surface charge of the sludge particles by charge neutralization and interparticle bridging, resulting in particle destabilization and aggregation, they were not able to break EPS and reduce bound water in sludge flocs^14^. However, Fenton treatment was more effective in converting bound water into free water by destructing sludge EPS with OH · oxidation, and consequently improving solid content of sludge cake.

#### Influence of chemical conditioning on EPS properties

**Figure 2:**
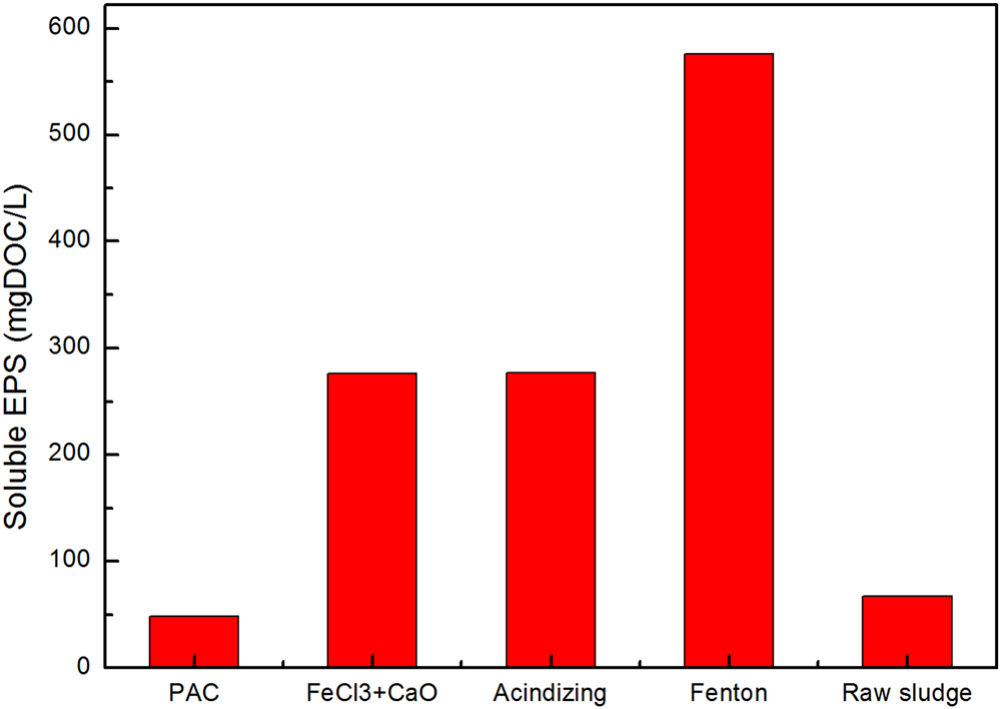
Effect of four sludge dewatering methods on solution EPS.

EPS are considered to be one of key constituents and affect physicochemical and biological properties of activated sludge system. They are also mainly responsible for sustaining the structural and functional integrity of the aggregates. EPS accounted for 60–80% of the mass of waste activated sludge, they play important roles in the removal of pollutants from wastewater, bioflocculation, settling and dewatering of activated sludge^20, 21, 28^. Figure 2 showed concentrations of SEPS were increased after treatment with different chemical conditioners. The concentrations of SEPS were 575.80 mg DOC/L, 277.40 mg DOC/L, 276.20 mg DOC/L, and 67.28 mg DOC/L under chemical conditioning with Fenton, acidification, FeCl_3_ + CaO and PAC respectively. As mentioned above, sludge particles were always negatively charged due to the ionization of anionic functional groups, such as carboxyl, amino and phosphate groups and so on. The presence of negative charge on the surface of particles could produce electronic repulsion and keep stability of a colloidal system. Inorganic coagulants could result in destabilization and aggregation of sludge particles by charge neutralization and bridging, and SEPS transferred from sludge bulk into solid phase through complex adsorption action of their hydrolyzed products^15^.

For Fenton treatment, ferrous could react with hydrogen peroxide under acidic conditions to generate highly reactive hydroxyl radicals which were able to quickly, efficiently solubilze and degrade organic matters in sludge system. Neyens *et al*.^29^ reported that Fenton reagent showed favourable cell-lysing effects and EPS dissolution, prompting water release. Acidic treatment could not only destroy EPS structure but caused protonation of anionic functional groups of EPS and aggregation of sludge system. Protein-like substances of low MC were solubilized and release into sludge bulk in acidic environment, consequently leading to increase of SEPS concentration. Additionally, under FeCl_3_ and lime conditioning, EPS dissolution can be attributed to the increases in proteins solubility and negative charge on the surface of sludge particles due to unprotonation of EPS at strong alkaline conditions, consequently decreasing the binding strength of EPS^30^.

3D-EEM spectroscopy has been used as an effective method to characterize EPS composition in wastewater treatment systems^31^. Each 3D-EEM spectrum is able to accurately respond the chemical compositions of EPS^17, 32^. Chen *et al*.^17^ had proposed a semi-quantification method using 3D-EEM base on fluorescence region integration (FRI). According to the peaks located at the excitation/emission wavelengths (Ex/Em), aromatic protein-like substances (APN), tryptophan protein-like substances (TPN), humic acids (HA) and fulvic acids (FA) were indicated respectively^33^. According to TOC and 3D-EEM data, the concentration of different organic substances in EPS could be obtained.

**Figure 3:**
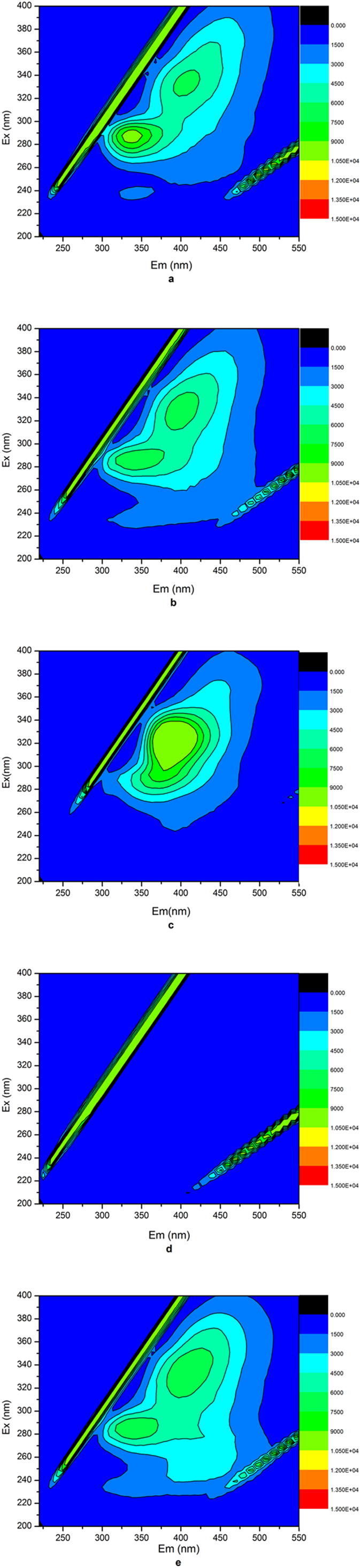
SEPS 3D-EEM fluorescence spectra under sludge conditioning.

As shown in Fig. 3, the main peaks for the SEPS were located at the excitation/emission wavelengths range of 260–300/300–375 nm and 300–380/375–475 nm, corresponding to TPN and HA, respectively. It was obvious that matters were dominated by humic substances in SEPS. Zhang *et al*.^16^ reported that the dominant composition of SEPS changed with the seasons, humic substances (HS) dominated in summer and protein-like matters in winner. HA was polymer organic acid consisting of aromatic and multiple functional groups and always originated from microbial decomposition of organic matters. FA contained significant amounts of phenolic hydroxyl and carbonyl groups which would react with oxides and metal ions. From Fig. 3, the fluorescent intensities of both TPN and HA were slightly decreased under PAC conditioning, and the fluorescent intensities of both TPN and HA were obviously decreased after conditioning with Fenton treatment. It was because proteins and HA were both removed by complex adsorption with hydrolyzed products of PAC. So the concentrations of SEPS with PAC were reduced in comparison to that of raw sludge. For Fenton treatment, ferrous could react with hydrogen peroxide under acidic conditions to generate highly reactive hydroxyl radicals which were able to quickly, efficiently solubilze and degrade organic matters in sludge system. Neyens *et al*.^29^ reported that Fenton reagent showed favourable cell-lysing effects and EPS dissolution, prompting bound water release. Acidic treatment could not only destroy EPS structure but caused protonation of anionic functional groups of EPS and aggregation of sludge system. Protein-like substances of low MC were solubilized and release into sludge bulk in acidic environment, consequently leading to increase of SEPS concentration. Additionally, under FeCl_3_ and lime conditioning, EPS dissolution could be attributed to the increases in proteins solubility and negative charge on the surface of sludge particles due to unprotonation of EPS at strong alkaline conditions, consequently decreasing the binding strength of EPS. And there was a strong binding strength between iron ions and protein, and protein transferred from SEPS to BEPS by the coagulation effect of iron ions in high pH value^30, 34^.

### Effects of different chemical conditioners on the odorous pollutants emissions

#### Inorganic gaseous pollutants

It is well known that H_2_S gas and NH_3_ gas come from biochemical reactions of inorganic salts and organic substances containing sulphur and nitrogen. Sulfate ions can be converted into H_2_S by sulfate-reducing bacteria (SRB) under anaerobic conditions. SRB can utilize the low molecular-weight organic matters as the carbon/energy substrates which are oxidized either completely to CO_2_ and/or some intermediate compounds using sulfate as a terminal electron acceptor, consequently generating sulphide (Eq. 1)^35^.

1 $${\rm{Organic}}\,{\rm{matter}}+{{\rm{SO}}}_{4}^{2-}\to {{\rm{HS}}}^{-}+2{{\rm{HCO}}}_{3}^{-}+{{\rm{CH}}}_{3}{{\rm{OO}}}^{-}$$

Moreover, there are other common microbial actions in the nitrogenous compounds (such as nitrate respiration and ammonification). Variety of bacterial and fungal use nitrate which exist in sludge and sludge liquor as the final receptor oxidized organic compounds and energy source, due to nitrate reductase (NR) is played the important role (Eq. 2). The reaction form and physiological effect are the similar to aerobic respiration. It is a far more efficient than fermentation. Following denitrification, non-nitrogen organic compounds were oxidized (Eq. 3). Many bacteria, gram-negative nonspore-bearing bacillus, can cause denitrification (such as fluorescent bacteria, stu perczel pylori). These are usually facultative aerobic bacteria, and denitrification often occurs under anaerobic condition. And some chemical autotrophic bacteria can also cause denitrification under anaerobic condition. For example, denitrifying sulfur bacteria use nitrate to oxide sulfur and reduce nitrate (Eq. 4). SRB also use nitrogen in amino acid as nitrogen source on one condition. A few SRB get nitrogen through dissimilatory reduction reaction from nitrate and nitrite. Ammonification is also called deamination. It is a degradation process of organic nitrogen compounds in the ammonification microorganisms (e.g. Bacillus, Clostridium difficile Bacillus and single spore bacteria, etc.), then NH_3_ release (Eq. 5). It was reported that Bacillus played a strong role on ammonification of organic nitrogen. And nitrite is also reduced to NH_4_^+^ by alienation of nitrite reduction bacterium (Eq. 6).

2 $${{\rm{NO}}}_{3}^{-}+2{{\rm{H}}}^{+}\to {{\rm{HNO}}}_{2}+{{\rm{H}}}_{2}{\rm{O}}$$

3 $${{\rm{C}}}_{6}{{\rm{H}}}_{12}{{\rm{O}}}_{6}+4{{\rm{NO}}}_{3}^{-}\to 6\,{{\rm{CO}}}_{2}\uparrow +6\,{{\rm{H}}}_{2}{\rm{O}}+2\,{{\rm{N}}}_{2}\,\uparrow $$

4 $$5\,{\rm{S}}+6\,{{\rm{NO}}}_{3}^{-}\to 3\,{{\rm{SO}}}_{4}^{2-}+3\,{{\rm{N}}}_{2}\,\uparrow $$

5 $${\rm{Organic}}\,{\rm{nitrogen}}\to {{\rm{NH}}}_{4}^{+}\to {{\rm{NH}}}_{3}\,\uparrow $$

6 $${{\rm{NO}}}_{2}^{-}\to {{\rm{NH}}}_{4}^{+}\to {{\rm{NH}}}_{3}\,\uparrow $$

**Figure 4:**
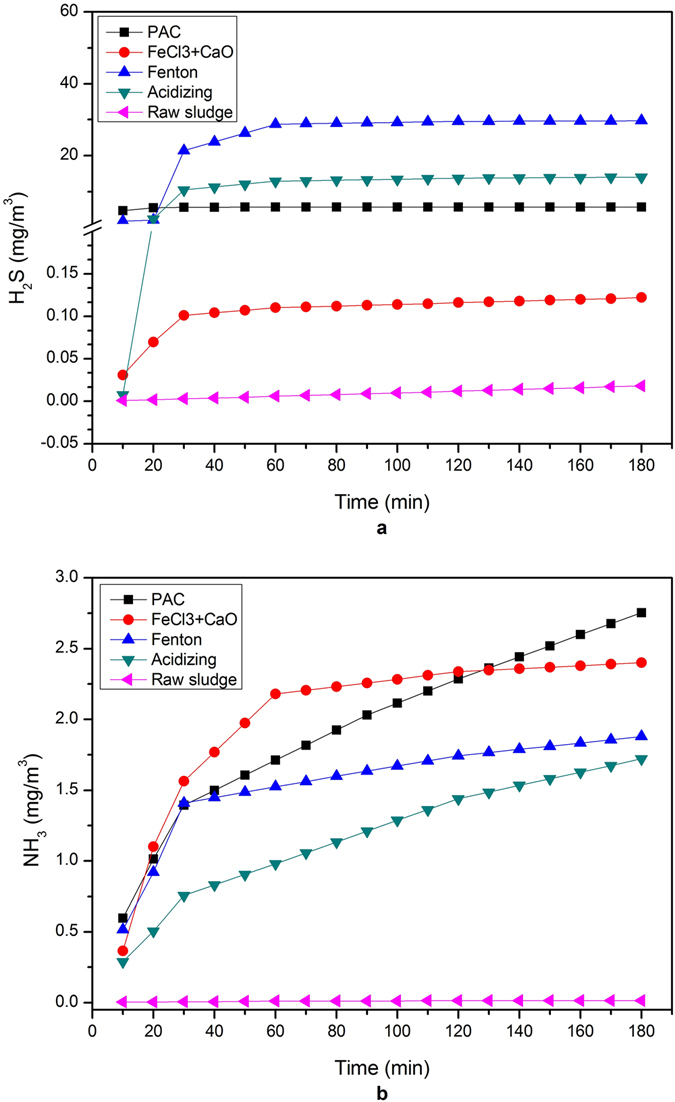
Effect of four sludge dewatering methods on the concentration of H2S (**a**) and NH3 (**b**) release.

As described in Fig. 4(a), the amount of H_2_S release increased gradually with dosages of varied inorganic coagulants adding. Hydrogen sulfide in absorption solutions were determined by simplified methylene blue spectrophotometric method. It can be seen that the varied inorganic coagulants demonstrated a positive promoting role in the H_2_S release process. The contents of H_2_S released under chemical conditioning were as follows: Fenton > Acidizing > PAC > FeCl_3_ + CaO. As described in Fig. 4(b), the amounts of NH_3_ release increased gradually with dosages of inorganic coagulants. NH_3_ concentration in sludge liquor was determined by sodium hypochlorite-salicylic acid method. It can be seen that the contents of NH_3_ release were as follows: PAC > FeCl_3_ + CaO > Fenton > Acidizing. Moreover, NH_3_ emission was much lower than the H_2_S emission. Allen *et al*. concluded that emissions of NH_3_ during the normal operation of waste water treatment plants had not been considered as a significant source^35^. Battye *et al*.^36^ also reported that the sewage treatment plant was unapparent source of NH_3_ emissions. However, NH_3_ emission was significantly intensified under chemical conditioning processes.

In the Fenton conditioning, pH value was firstly adjusted to 3. It was because the conditioning effect of Fenton mainly achieved balance when the pH value was less than 4. Then Fe^2+^ could form FeS precipitates with sulfide (Eq. 7).

7 $${{\rm{Fe}}}^{2+}+{{\rm{HS}}}^{-}\to {\rm{FeS}}\downarrow +{{\rm{H}}}^{+}$$

Low pH value was conducive to inhibit the formation of FeS precipitates. It prompted H_2_S to release from sludge into air^35^. Moreover, Yuan *et al*.^37^ reported that ferrous irons could significantly improve the activity of activated sludge microorganisms by accelerating electron transfer and function as enzyme activators, thus improving organic matters degrading, so NH_3_ and HS^−^ were produced. Acidizing method only accelerated the forward reaction of Eq. 8, and H_2_S gas quickly released from sludge solution. So pH value was increased rise slightly due to emission of H_2_S (Figure S1). However, acidizing treatment inhibited the NH_3_ releasing due to formation of NH_4_^+^ through protonation, and the amount of NH_3_ release was lower in comparison to other chemical reagents.

8 $${{\rm{HS}}}^{-}+{{\rm{H}}}^{+}\to {{\rm{H}}}_{2}{\rm{S}}\,\uparrow $$

Hydrolysis reaction of Al^3+^ formed aluminum hydroxide (Al(OH)_3_) deposition which was able to neutralize negative surface charge of sludge particles and promote their aggregation. As shown in the Eq. 9, S^2−^ in solution can enhance hydrolysis reaction of Al^3+^, and H_2_S was released at the same time. Then PAC was the most commonly inorganic polymer flocculants and was characterized of hydrolysis stability. So pH value of slurry changed slightly and the concentration of H_2_S released was lower than acidizing and Fenton treatment. In neutral conditions, the positive reaction of Eq. 8 was weak. The concentration of H_2_S release was quite up to the balance (Fig. 4(a)). Besides, there was a little bit of influence by the microorganism. Al(OH)_3_ flocculation caused hypoxia inside of microorganism. It was because anoxic condition formed with the increasing of oxygen transfer resistance and consumption of external aerobic bacteria. So advantage microbial was nitrifying bacteria. Then NH_3_ emissions decreased. Burton^38^ had studied that the nitrogen lost to the atmosphere as ammonia was reduced under anaerobic conditions.

9 $$2{{\rm{Al}}}^{3+}+3{{\rm{S}}}^{2-}+6{{\rm{H}}}_{2}{\rm{O}}\to 2{\rm{Al}}{({\rm{OH}})}_{3}\downarrow +3{{\rm{H}}}_{2}{\rm{S}}\,\,\uparrow $$

Fe^3+^ directly formed Fe_2_S_3_ precipitates with sulfide, which facilitated ionization of H^+^. S^2−^ could be oxidized to S precipitate by Fe^3+^ (Eq. 10). Then Fe_2_S_3_ transformed into FeS. With addition of lime (CaO), pH value rised to above 10, which greatly promoted NH_3_ release from sludge. Liu *et al*.^39^ reported that emission of NH_3_ increased with increase in lime dosage.

10 $${{\rm{Fe}}}^{3+}+2{{\rm{HS}}}^{-}\to {\rm{FeS}}\downarrow +{\rm{S}}\downarrow +2{{\rm{H}}}^{+}$$

#### VOCs emission

**Figure 5:**
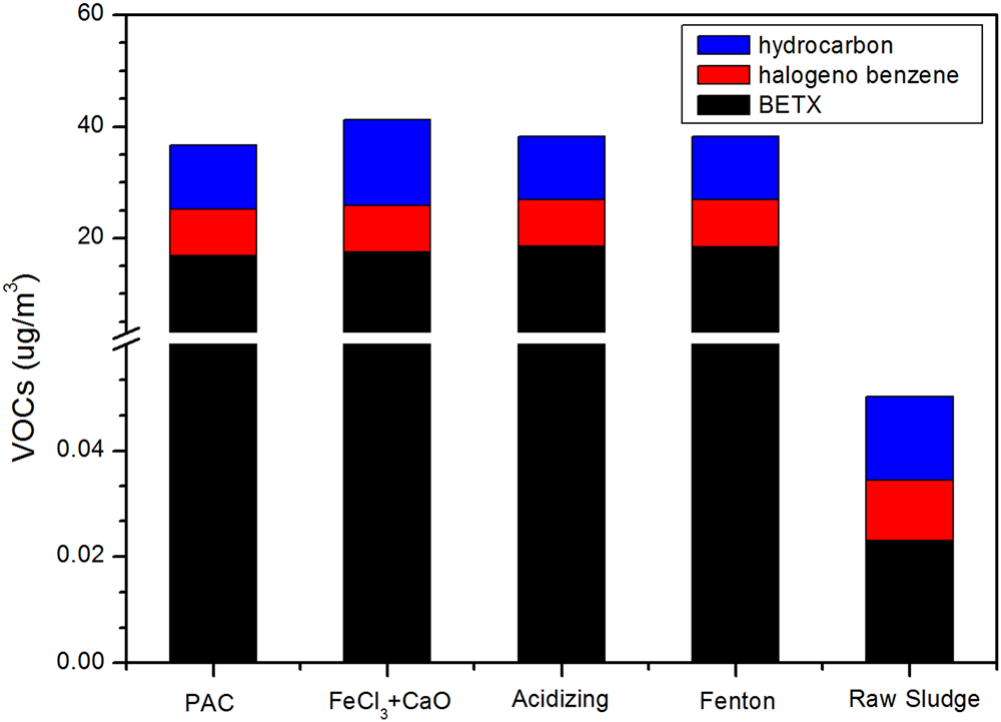
Effect of chemical conditioning on VOCs emissions divided into three main groups compositions.

They always come from petrochemical wastewater, coking industry wastewater, pesticide wastewater, pharmaceutical wastewater, etc. Thus, VOCs was mainly composed of organic compounds containing benzene and hydrocarbons. VOCs are hydrophobic substances with low water solubility, they are inclined to adsorb on extracellular polymeric substance (EPS) through hydrophobic interactions. As described in Fig. 5, VOCs emission was evidently increased under different chemical conditioning processes. The total VOCs concentrations were 0.05 ug/m^3^, 36.70 ug/m^3^, 41.22 ug/m^3^, 38.24 ug/m^3^ and 38.30 ug/m^3^ for raw sludge, PAC, FeCl_3_ + CaO, acidizing and Fenton treatment, respectively.

**Figure 6:**
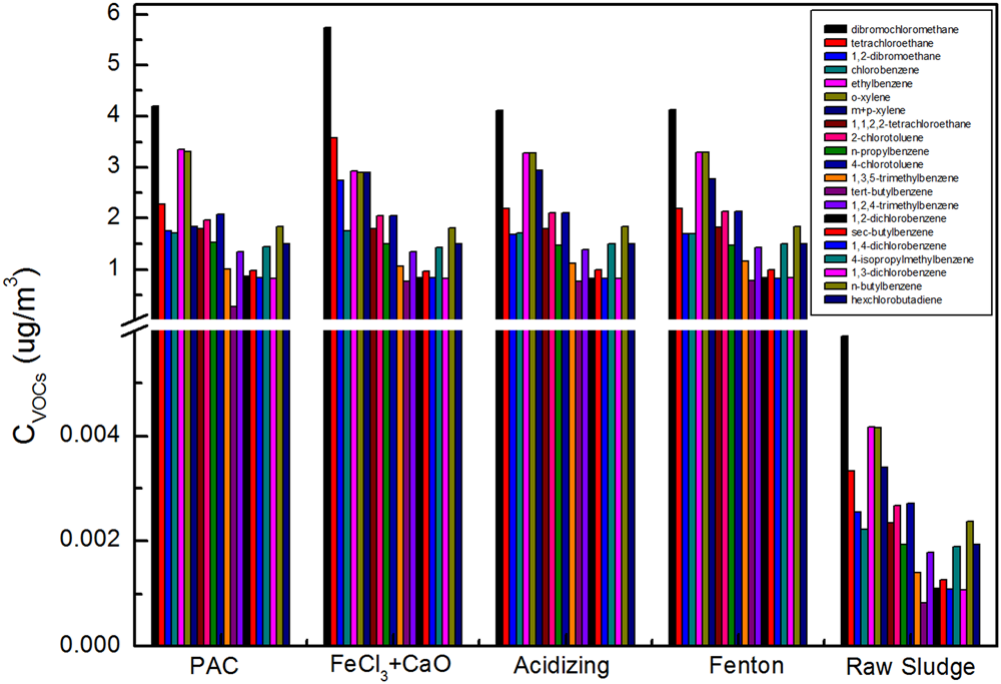
Composition analysis of VOCs.

As shown in the Fig. 6, there were 21 VOCs could be detected by GC-MS. Dibromochloromethane, tetrachloroethane, 1,2-dibromoethane, ethylbenzene, o-xylene, m-xylene and p-xylene were the main compounds in VOCs. And dibromochloromethane emission was the maximum under FeCl_3_ + CaO conditioning.

VOCs emissions were generally classified into three main compositions: benzenes, halogeno benzenes and hydrocarbons. As can be seen from Fig. 5, benzenes were 18.48 ug/m^3^ and 18.53 ug/m^3^ for Fenton and acidizing treatment, which was higher than other two conditioning processes. There was no obvious difference in the amounts of halogeno benzene released for four conditioning methods. Hydrocarbons emission in FeCl_3_ + CaO conditioning was higher than that under other three conditioning processes.

#### Mass balance of odorous pollutants

**Figure 7:**
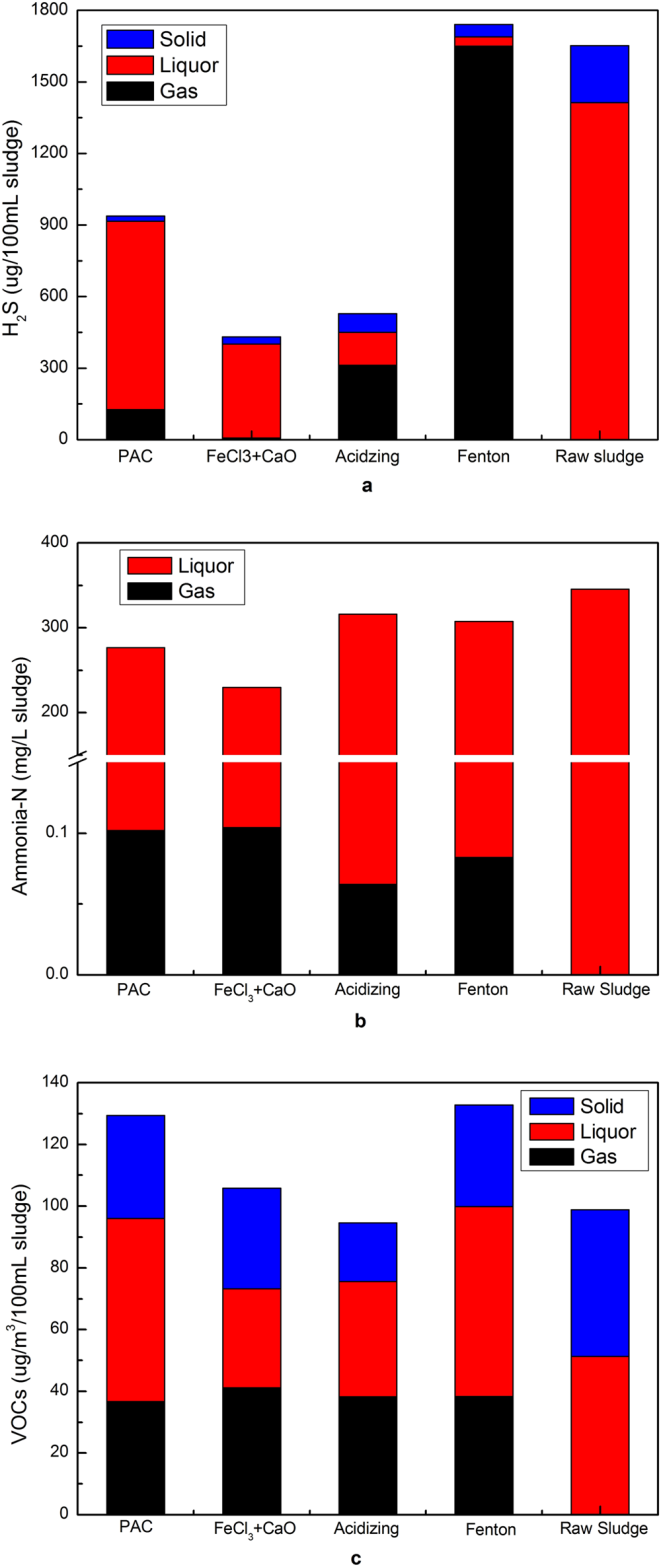
Mass balance of odorous pollutants under different conditioning processes (**a**) H_2_S; (**b**) Ammonia-N; (**c**) VOCs.

The concentrations of H_2_S, ammonia nitrogen (Ammonia-N) and VOCs in the condition of solid, liquor and gas were analyzed. From Fig. 7(a), the concentration of H_2_S in the liquor phase of raw sludge was obviously much more than it in the solid phase. The concentration of H_2_S in the liquor phase with PAC and FeCl_3_ + CaO conditioning was also more than that treated with acidizing and Fenton conditioning. Compared with raw sludge, the concentrations of H_2_S in the liquor phase and solid phase both drastically reduced. And the concentration of H_2_S in the gas phase with acidizing and Fenton treatment was higher than that conditioned with PAC and FeCl_3_ + CaO conditioning. As shown in Fig. 7(a), it indicated a migration process of sulfur (S) from solid to liquor, and to air lastly. In the liquor phase and solid phase, Ammonia-N was used to represent nitrogen (N) content. As described in Fig. 7(b), there was no ammonia-N detected in the solid phase. The concentration of ammonia-N in the liquor phase significantly decreased after sludge conditioning. The concentrations of NH_3_ emission in the gas phase were higher under PAC and FeCl_3_ + CaO treatment, and while NH_3_ concentration were higher in the liquor phase after conditioning with acidizing and Fenton. It was very likely that protonation of NH_3_ inhibited its release at acidic conditions. As shown in Fig. 7(c), the concentrations of VOCs in the liquor phase had no significant changes in raw sludge, PAC and Fenton conditioning. And yet the concentrations of VOCs in the solid phase greatly were reduced under FeCl_3_ + CaO and acidizing conditioning. It was because that VOCs was generally hydrophobic and adsorbed on slurry by EPS by hydrophobic and electrostatic interactions. Then VOCs released when EPS was destroyed. The total VOCs emission was highest under Fenton conditioning, it was minimum under acidizing treatment. It was reported that VOCs emissions were influenced by variations of temperature^40, 41^, oxygen^42^, pH^43^ and EPS properties^33^. However, pH value of sludge was significantly changed under different conditioning (see in the Figure S1). As mentioned above, pH value was one of most important factors affecting odours emission in the process of sludge treatment. The major mechanism of flocculation conditioning was EPS densification, VOCs emission was the minimum with PAC. Acidific treatment resulted in solubilization of complexes of EPS and cations^29^, so the concentration of SEPS increased and VOCs released at the same time. It was because the concentration of SEPS not only reflected the destruction extent of EPS with chemical reagents, but also indirectly reflected the adsorption of EPS on VOCs. Fenton treatment could effectively destroy EPS fraction through oxidation process, consequently cause emission of a large amounts of VOCs. From Fig. 7(a–c), H_2_S and NH_3_ released were mainly originated from liquor phase, while VOCs came from solid and liquid phases. Obviously, there were significant differences in total mass of odorous pollutants before and after conditioning, especially for H_2_S. A portion of sulfide ions were oxidized into S_0_/sulfate ions under Fenton treatment, while they were able to form metal sulfur precipitates with addition of ferric ions. However, the contents of element sulfur and metal sulfur precipitates in sludge particles were rather difficult to be determined, leading to discrepancy in mass balance of different odorous pollutants. In addition, Fenton treatment also might result in VOCs degraded.

## Conclusions

The study investigated transfer behavior of odorous pollutants in wastewater sludge system under typical chemical conditioning processes for dewaterability enhancement. A considerable amount of research work has been carried out on the relationships between sludge dewaterability and sludge characteristics, but few of them has reported emission of odorous pollutants under chemical conditioning. This study indicated that a large amounts of odorous pollutants (H_2_S, NH_3_ and VOCs) were released a under different conditioning processes. There were significant differences in composition and concentration of odorous pollutants at various treatments. The released contents of H_2_S were 29.70 mg/m^3^, 14.05 mg/m^3^, 5.74 mg/m^3^ and 0.12 mg/m^3^, while that of NH_3_ were 1.88 mg/m^3^, 1.72 mg/m^3^, 2.75 mg/m^3^ and 2.40 mg/m^3^ under chemical conditioning with Fenton, acidification, PAC, FeCl_3_ + CaO, respectively. Different conditioning processes could result into VOCs emission by affecting sludge solution chemistry conditions and EPS properties. The total VOCs concentrations were 36.70 ug/m^3^, 41.22 ug/m^3^, 38.24 ug/m^3^ and 38.30 ug/m^3^ for PAC, FeCl_3_ + CaO, acidizing and Fenton, respectively. PAC was ineffective to solubilize sludge EPS and thus VOCs emission was the minimum among four conditioning processes. Fenton and acidific treatment could effectively destroy EPS fraction, consequently cause emission of a large amounts of VOCs. H_2_S and NH_3_ released were mainly originated from liquor phase, while VOCs came from solid and liquid phases. This work provided a scientific and technical support for odors control in the process of chemical conditioning.

## Materials and Methods

### Materials

#### Waste sludge

Sludge was obtained from sludge thickening tank in Xiaohongmen wastewater treatment plant, Beijing, China. It treats approximately 600,000 m^3^ of wastewater daily, and is treated by Anaerobic–Anoxic–Oxic (A/A/O). Samples were stored at 4 °C and were analyzed within 7 days after sampling. The characteristics of the sludge are listed in Table 1. As shown in the Table 1, the raw sludge was bad dewaterability because the SRF value of the raw sludge was 37.21 E + 12 m · kg^−1^. The moisture content of sludge was 98.69%. The value of pH was 7.01, and zeta poteitial of sludge was −15.07 mV. From the testing of TOC analyzer, the SEPS of the raw sludge was 67.29 mg DOC/L.

**Table 1:** Characteristics of waste sludge.

Indicator	Moisture content (%)	pH	Zeta poteitial (mV)	TOC (mg/L)	SRF (E + 12 m/kg)
Value	98.69	7.01	−15.07	67.29	37.21

#### Chemical agents

The reagents used in this study were of analytical grade and purchased from Sinopharm Group Chemical Reagent Co., Ltd, such as FeCl_3_, CaO, H_2_SO_4_, H_2_O_2_ and FeSO_4_. PAC was produced by a local factory (Beijing global water science and technology Co. Ltd, China).

### Jar test

**Figure 8:**
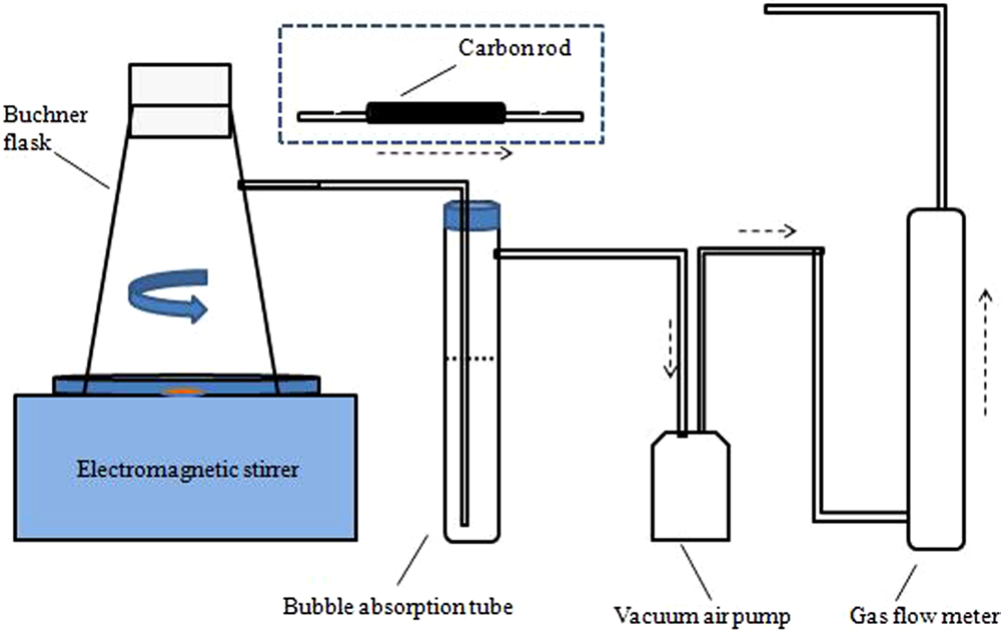
Experimental device of sludge treatment and odorous acquisition.

Jar tests were conducted on a programmable jar test equipment (Daiyuan Jar Test Instruments, China). Sludge samples were reacted with coagulants by using magnetic stirrer, and tester was started at rapid mixing of 200 rpm, different chemical conditioners were quickly added. Meanwhile, inorganic gaseous pollutants (H_2_S and NH_3_) and VOCs were gathered by solution absorption in the bubble absorption tube and activated carbon tube respectively. Figure 8 showed the experimental device of sludge conditioning and odorous contaminants collection. Note that the dosages of different chemical reagents used in this work were base on our previous studies.

In addition, sludge sample was settled down at 100 g for 15 min, and the supernatant (bulk solution) was collected as SEPS. Cellulose acetate (CA) membranes with a pore size of 0.45 µm were used to remove the particulates present in the sludge supernatant. The filtered fractions were used for analyzing fluorescence EEM and dissolved organic carbon (DOC).

### Analytical methods

#### Odorous pollutants analysis

H_2_S was measured by methylene blue spectrophotometric method (GB/T 11742–1989). NH_3_ was determined according to sodium hypochlorite-salicylic acid method (GB/T 14679–1993). GC-MS (6890GC/5973MSD, U.S.A, Agilent Co.) is used for VOCs analysis.

#### SRF

SRF was measured with the standard Buchner funnel test using a quantitative filter paper. It can be obtained by Eq. (11):

11 $$r=\frac{2P{A}^{2}b}{\mu \omega }$$

Where P (kg m^−2^) denotes pressure, A (m^2^) is filtration area, µ (kg s m^−2^) is kinetic viscosity, w (kg m^−3^) denotes dry solid weight per unit volume sludge on the filtrate media, b is slope of filtration equation t/V = bV + a, and t (s) is time, V (m^3^) denotes volume of filtrate. The raw or conditioned waste sludge was poured into a Buchner funnel with a 0.45 µm filter paper to filter under a pressure of 0.6 MPa with vacuum pump. Weight of filtrate was recorded every 10 s before surface cracking was observed. The equipment was shown as Figure S2.

#### Soluble EPS analysis

The sample was diluted with Milli Q water until concentration of DOC was below 10 mg/L. The peak locations, peak intensities and the ratios of different peaks in EEM spectra of the EPS samples were not substantially influenced by ionic strength^25^. Three dimensional excitation emission matrix (3-DEEM) spectra were measured by a Hitachi F-4500 fluorescence spectrophotometer with an excitation range from 200 to 400 nm at 10 nm sampling intervals and an emission range from 220 to 550 nm at 10 nm sampling interval. The spectra were recorded at a scan rate of 12,000 nm/min, using excitation and emission slit bandwidths of 10 nm. Each scan had 37 emission and 27 excitation wavelengths.

#### Other analytic methods

The dissolve organic carbon (DOC) of SEPS was analyzed using TOC analyzer (Shimadzu, Kyoto, Japan). pH was measured by a pHS-3C (Shanghai, China) pH meter, which was calibrated using pH 4.01, pH 7.01 and pH 9.18 buffers. Zeta potential of sludge was analyzed using zeta potential and nano/submicron particle size Analyzer (ZetaPALS, Malvern, UK).

## Electronic supplementary material

Supporting information
