# Supplementary material for: Transfer behavior of odorous pollutants in wastewater sludge system under typical chemical conditioning processes for dewaterability enhancement
Source: Sci Rep. 2017 Jun 13;7:3417. doi: 10.1038/s41598-017-03727-4 (PMC5469821; doi:10.1038/s41598-017-03727-4)
Supplement: Supplementary file 1 — Supporting information [file 41598_2017_3727_MOESM1_ESM.doc]

**Supporting information**

**Transfer behavior of odorous pollutants in wastewater sludge system under typical chemical conditioning processes for dewaterability enhancement**

Hongyu Gao^1,3^, Weijun Zhang^2,*^, Zhenzhen Song^4^, Xiaofang Yang^3^, Lian Yang^1^,

Mengdi Cao^1,3^, Dongsheng Wang^1,3,**^, Guiying Liao^1^

1. Faculty of Materials Science and Chemistry, China University of Geosciences, Wuhan 430074, Hubei, China
2. School of Environmental Studies, China University of Geosciences, Wuhan 430074, Hubei, China
3. State Key Laboratory of Environmental Aquatic Chemistry, Research Center for co-Environmental Sciences, Chinese Academy of Sciences, Beijing 100085, China
4. Technology Center, ZhongYang steel Co., Ltd, Zhongyang 033400, Shanxi, China

*** Corresponding Author 1**: Name: Weijun Zhang, Email: [zhwj_1986@126.com](mailto:zhwj_1986@126.com)

**** Corresponding Author 2**: Name: Dongsheng Wang, Email: wgds@rcees.ac.cn

**Figure S1 pH**


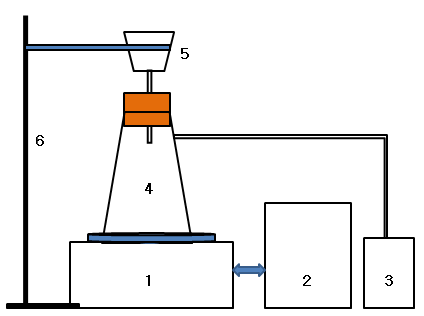


**Figure S2 SRF equipment**

**(1. Electronic scales 2. Computer 3. Vacuum air pump**

**4. Buchner flask 5. Buchner funnel 6. Iron support)**
